# Supplementary figures and images for: Omega-3 fatty acid normalizes postsynaptic density related miRNAs and proteins in hippocampus and prevents DEHP-induced impairment of learning and memory in mice
Source: PLoS One. 2025 Jul 3;20(7):e0313233. doi: 10.1371/journal.pone.0313233 (PMC12225789; doi:10.1371/journal.pone.0313233)

Western blot Fig.6

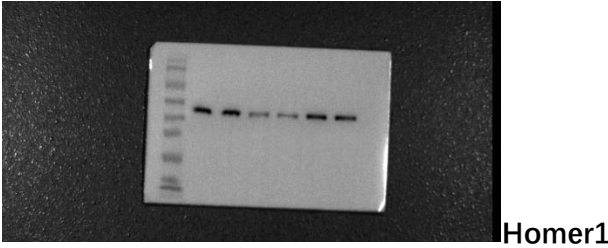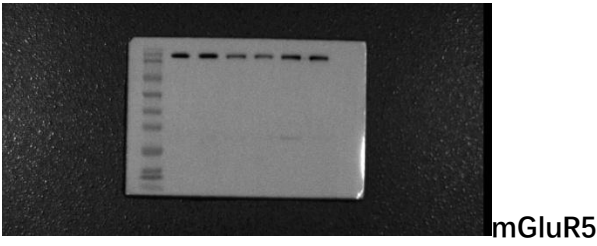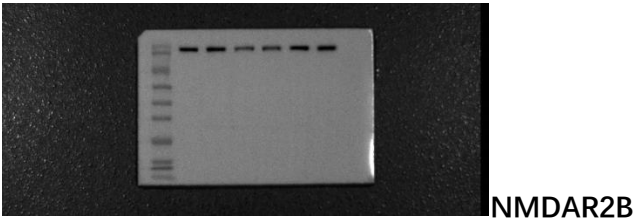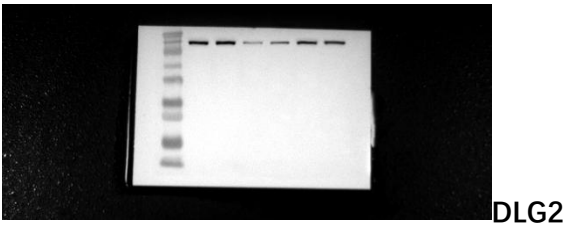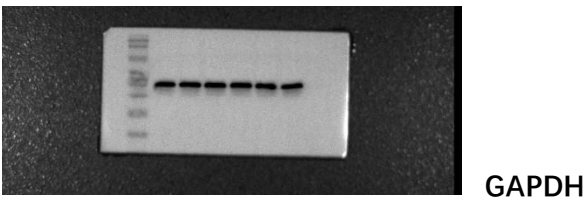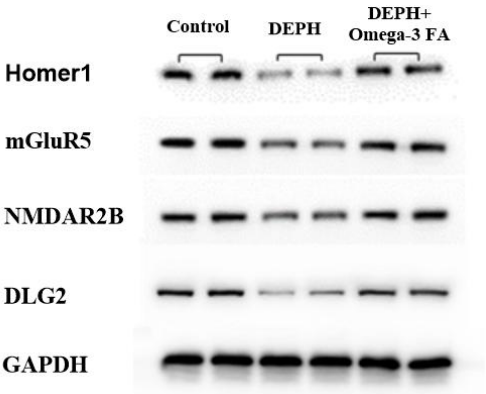

Supplement: S1 — (PDF) [file pone.0313233.s001.pdf]
