# Supplementary material for: Omega-3 fatty acid normalizes postsynaptic density related miRNAs and proteins in hippocampus and prevents DEHP-induced impairment of learning and memory in mice
Source: PLoS One. 2025 Jul 3;20(7):e0313233. doi: 10.1371/journal.pone.0313233 (PMC12225789; doi:10.1371/journal.pone.0313233)
Supplement: Attachment — Submitted filename: Response to Reviewers.docx [file pone.0313233.s004.docx]

**The responses to the reviewers’ comments are as following:**

**Reviewer #1:** This work compared the learning and memory abilities, PSD microstructures, miRNA expression profiles and PSD-related proteins of the mice treated with DEHP or DEHP+Omega-3FA. They concluded that Omega-3FA protected DEHP-induced impairment of learning and memory as well as synaptic structure alteration in the hippocampus by regulating the expression of PSD associated miRNAs and their targets. This manuscript is well written. The findings regarding the beneficial effects of Omega-3FA on the DEHP-induced impairments of memory and PSD structure are novel and interesting. However, the contributions of the molecular changes to behavioral and cellular changes are poor, which can not support their conclusion described as "... by regulating the expression of PSD associated miRNAs and their targets". Some major issues are as follow:

**Response:** Thank you very much for reviewing our manuscript and giving us so many useful suggestions. Your comments have helped us to improve our article significantly. We highlighted all changes in yellow in our revised manuscript.

**Comment 1：**Changes of some miRNAs and proteins were observed in the hippocampi of the mice with DEHP or DEHP+Omega-3FA treatment. There lacks experimental evidence to support the regulation of PSD proteins by those changed miRNAs. In addition, a causal relationship between differentially expressed (DE)-miRNAs and PSD structure alteration is not determined.

**Response:** Thank you for your helpful suggestion. We are also willing to verify the relationship between these miRNAs and their target proteins, but currently our research mainly focuses on screening them. In the future, we will conduct in-depth research on the regulation between important miRNAs and their target proteins. As your suggestion, the relationship between DE-miRNAs and PSD structure is not determined, the FISH assay should be performed, however, we have not yet successfully completed the FISH of miRNA, we would like to adopt your suggestions as our technology improves in the future. Furthermore, we also corrected “postsynaptic density proteins via miRNAs regulation” to “postsynaptic density related miRNAs and proteins” in the title and article.

**Comment 2：**I suggest that the levels of some important DE-miRNAs between these groups should be compared by qPCR experiments, and some of them may also be confirmed to localized at synapses by fluorescence in situ hybridization (FISH) method.

**Response:** Thank you for your helpful suggestion. As your suggestion, the relationship between DE-miRNAs and PSD structure is not determined, the FISH assay should be performed, however, we have not yet successfully completed the FISH of miRNA, we would like to adopt your suggestions as our technology improves in the future. The levels of four important miRNAs (Three or more PSD related proteins are regulated in bioinformatics analysis) were selected and confirmed using qPCR (Figure 4), which is consistent with the results of the miRNA sequencing.

**Comment 3：**Only DE-miRNAs were shown in the present version, additional data regarding miRNA sequencing should be prepared as supplementary files.

**Response:** Thank you for your helpful suggestion. The additional data regarding miRNA sequencing has been uploaded as supplementary files in our revised manuscript.

**Comment 4:** As described in method section, they made GO function and KEGG pathway analyses using the identified DE-miRNAs. Some enriched GO funtions (Cellular Component) were listed in Table 1. Where is the data from the enriched KEGG pathways?

**Response:** Thank you for your helpful suggestion. We are sorry for including “KEGG pathway analyses” in the experimental method, but we only used Go function in reality. We have corrected this mistake in our new version.

Reviewer #2: In the present study, authors have investigated the potential effect of omega-3 fatty acids on DEHP-induced neurotoxicity in mice using molecular and behavioral methods. Overall, the subject is timely and interesting, methodology is solid and results are clear to the readers, however, I propose the following concerns to be addressed prior to publication of this research:

**Response:** Thank you very much for reviewing our manuscript and giving us so many useful suggestions. In our new manuscript, we have corrected all the issues according to your suggestions. We have added a flowchart to the article, reanalyzed the behavioral results, and organized the structure of the introduction and discussion section, etc. We hope that our manuscript will better.

Major concerns:
1. Ethical considerations/protocols must be clarified in the manuscript (ethics code/number etc.)

**Response:** Thank you for your helpful suggestion. Ethical considerations have been clarified in our revised manuscript.

2. Please describe the combination of O3 fatty acids in details (type and proportion of fatty acids used in the study). By the way, dose of O3 (150 mg/kg) should be justified reasonably in discussion.

**Response:** Thank you for your helpful suggestion. We have provided the type and proportion of omega-3 (18% EPA, 12%DHA)[1, 2] in the paragraph of animal treatment in the revised manuscript.

Neuroprotective efficacy of Omega-3 FA has been studied at different doses (100–1000 mg/kg b.wt.) in animal models of disease conditions and neurotoxicity [2-6]. The dose of Omega-3 FA (150 mg/kg) was based on the studies carried out by [Shaaban](https://pubmed.ncbi.nlm.nih.gov/?term=Shaaban+AA&cauthor_id=24144775) et al [6].

The human dose equivalent for the used animal dose of Omega-3 FA (150 mg/kg) is approximately 18 mg/kg [7], which meets the standard recommended daily doses for humans[8].

3. I strongly suggest to present the results of MWM during time (trial period in Days) and then re-analyze the results by two-way ANOVA. This would enable the reader to more deeply understand how memory function is affected by learning and treatments.

**Response:** Thank you for your helpful suggestion. We have reanalyzed escape latency using two-way ANOVA in our revised version, showing the dynamic changes of training and the results appear more complete. Thank you again.

4. I expect the authors to provide a detailed graphical timeline demonstrating the main experimental protocols in a sequential manner.

**Response:** Thank you for your helpful suggestion. We have provided our experimental protocols in a sequential manner in the revised manuscript (Fig.1A.).

5. In Introduction and/or Discussion, authors have ignored recent literature on the effect of omega-3 fatty acids on memory function (please see <https://doi.org/10.1016/j.lfs.2023.122100,> and <https://doi.org/10.1002/jdn.10336>).
**Response:** Thank you for your helpful suggestion. The two recent literatures on the effect of omega-3 fatty acids on memory function have been added in our revised introduction.

Minor concerns:
1. Please use “Control” instead “Con” in figures.

**Response:** Thank you for your helpful suggestion. We have changes “Control” instead “Con” in the revised manuscript.

2. The entire manuscript required revision for English (mainly consistency and clarity of Discussion).

**Response:** Thank you for your helpful suggestion. The manuscript has been revised for English.

Reviewer #3:

1. Introduction: This part is far too vague and contains too many generalities. There is a need to better explain.

**Response:** Thank you for your helpful suggestion. We have reorganized and revised the Introduction, we hope it will be better.

2. Please provide the volume of DEHP and Omega-3 FA injectate administered for gavage.
**Response:** Thank you for your helpful suggestion. The volume of DEHP and Omega-3 FA injectate administered for gavage is 1ml, was added in revised manuscript.

3. Did the control group receive normal saline gavage?

**Response:** Thank you for your helpful suggestion. Mice in control group received the same volume corn oil gavage, which was added in revised manuscript.

4. Western blotting: Write homogenization process in detail mentioning the amount of tissue homogenized, volume and pH of buffer used, temperature for homogenization and include the make and model of the homogenizer. How much protein was loaded on the gel? Mention incubation time with primary and secondary antibodies. It is advised to specify each antibody used (primary and secondary), its concentration and the provider.

**Response:** Thank you for your helpful suggestion. We have rewritten the part of Western blotting according to your suggestion in our revised manuscript.

Reviewer #4: The author was well written the manuscript. Further, I picked up on a few additional points for consideration and correction:
1. Did you use the behavioral assessment rats for further neurochemical studies or not? If you did not use these rats, then explain what happened with behavioral assessment rats.

**Response:** Thank you for your helpful suggestion. We didn’t perform the behavioral assessment rats for further neurochemical studies. We have explained our results in the revised manuscript as following: Morris water maze (MWM) performance is a standard technique in measure of hippocampally dependent spatial navigation and reference memory[9]. MWM test has been employed in numerous studies demonstrating cognitive ability following changes of learning and memory induced by neurotoxins in the environment or nutrients in food in rodents [10]. The escape latency to identify the hidden platform and the times of crossing in the target quadrant are collected as the parameter of learning function and memory retention, respectively. Though MWM test, we found that Omega-3FA could protect against DEHP-induced impairment of learning and memory.

2. Animal ethical committee approval number should be mention in the methodology section.

**Response:** Thank you for your helpful suggestion. Ethical considerations have been clarified in our revised manuscript.

3. Justification of DEHP doses is lacking in the manuscript, mention in the methodology section.

**Response:** Thank you for your helpful suggestion. The Justification of DEHP doses has been added in the revised manuscript. The concentration of DEHP exposure were set based on the [Reference Dose](http://www.baidu.com/link?url=w1bvagZHbQquF6hR1_k5VDHqbv2LTn9aMXJlH84em_Rh9BGQFd1s0L8iKHWOZdtCTKDkrnRKWwTskU9p5ixlpqIDpM302AElreiU0yo5EmlmZXO6ksiXrK9CCjAA1ET71p_pBNH2L7ZJyVVaG34uTa) (RfD) set by the Environmental Protection Agency (20 times of the R_f_D for children give cause for concern, and this dosage falls within the range of exposures previously documented in humans) [11-14].

4. PSD95 is interact with DLG2 proteins and having in the role of glutamate levels, author need to check the DLG2 expression levels as well as the glutamate levels.

**Response:** Thank you for your helpful suggestion. The DLG2 expression level has been measured, and this result has been added in Fig. 6 in our revised manuscript.

5. How much amount of DEHP have reached to brain, it has to be discussed about the levels of DEHP is responsible for these alterations in the brain.

**Response:** Thank you for your helpful suggestion. I’m sorry that our description in the discussion may have caused you a misunderstanding. We have searched a large number of literatures, however, there are no studies provided certain evidence to prove the levels of DEHP in brain, except some discussed DEHP can cross the BBB[15]. Therefore, we discussed in our revised manuscript and highlighted marked.

1.	Mori MA, Delattre AM, Carabelli B, Pudell C, Bortolanza M, Staziaki PV, et al. Neuroprotective effect of omega-3 polyunsaturated fatty acids in the 6-OHDA model of Parkinson's disease is mediated by a reduction of inducible nitric oxide synthase. Nutr Neurosci. 2018;21(5):341-51. Epub 2017/02/22. doi: 10.1080/1028415x.2017.1290928. PubMed PMID: 28221817.

2.	Elblehi SS, El Euony OI, El-Sayed YS. Apoptosis and astrogliosis perturbations and expression of regulatory inflammatory factors and neurotransmitters in acrylamide-induced neurotoxicity under ω3 fatty acids protection in rats. Neurotoxicology. 2020;76:44-57. Epub 2019/10/28. doi: 10.1016/j.neuro.2019.10.004. PubMed PMID: 31647937.

3.	Singh PK, Nath R, Ahmad MK, Rawat A, Babu S, Dixit RK. Attenuation of lead neurotoxicity by supplementation of polyunsaturated fatty acid in Wistar rats. Nutr Neurosci. 2016;19(9):396-405. Epub 2015/05/26. doi: 10.1179/1476830515y.0000000028. PubMed PMID: 26005885.

4.	El-Ansary AK, Al-Daihan SK, El-Gezeery AR. On the protective effect of omega-3 against propionic acid-induced neurotoxicity in rat pups. Lipids Health Dis. 2011;10:142. Epub 2011/08/23. doi: 10.1186/1476-511x-10-142. PubMed PMID: 21854591; PubMed Central PMCID: PMCPMC3170231.

5.	Singh PK, Singh MK, Yadav RS, Nath R, Mehrotra A, Rawat A, et al. Omega-3 fatty acid attenuates oxidative stress in cerebral cortex, cerebellum, and hippocampus tissue and improves neurobehavioral activity in chronic lead-induced neurotoxicity. Nutr Neurosci. 2019;22(2):83-97. Epub 2017/08/02. doi: 10.1080/1028415x.2017.1354542. PubMed PMID: 28760072.

6.	Shaaban AA, Shaker ME, Zalata KR, El-kashef HA, Ibrahim TM. Modulation of carbon tetrachloride-induced hepatic oxidative stress, injury and fibrosis by olmesartan and omega-3. Chem Biol Interact. 2014;207:81-91. Epub 2013/10/23. doi: 10.1016/j.cbi.2013.10.008. PubMed PMID: 24144775.

7.	Reagan-Shaw S, Nihal M, Ahmad N. Dose translation from animal to human studies revisited. Faseb j. 2008;22(3):659-61. Epub 2007/10/19. doi: 10.1096/fj.07-9574LSF. PubMed PMID: 17942826.

8.	Campisi SC, Zasowski C, Bradley-Ridout G, Schumacher A, Szatmari P, Korczak D. Omega-3 fatty acid supplementation for depression in children and adolescents. Cochrane Database Syst Rev. 2024;11(11):Cd014803. Epub 2024/11/20. doi: 10.1002/14651858.CD014803.pub2. PubMed PMID: 39564892; PubMed Central PMCID: PMCPMC11577543 Child and Adolescent Psychiatry (Chair, Research and Scientific Program Committee); Canadian Pediatric Society (Chair, Mental Health Task Force); Canadian Institutes of Health Research (CIHR) (Grant/Contract); Hospital for Sick Children (Grant/Contract); University of Toronto (Grant/Contract); Psychiatrist, Hospital for Sick Children.

9.	Vorhees CV, Williams MT. Morris water maze: procedures for assessing spatial and related forms of learning and memory. Nat Protoc. 2006;1(2):848-58. Epub 2007/04/05. doi: 10.1038/nprot.2006.116. PubMed PMID: 17406317; PubMed Central PMCID: PMCPMC2895266.

10.	Luo M, Li L, Ding M, Niu Y, Xu X, Shi X, et al. Long-term potentiation and depression regulatory microRNAs were highlighted in Bisphenol A induced learning and memory impairment by microRNA sequencing and bioinformatics analysis. PLoS One. 2023;18(1):e0279029. Epub 2023/01/20. doi: 10.1371/journal.pone.0279029. PubMed PMID: 36656826; PubMed Central PMCID: PMCPMC9851566.

11.	Koch HM, Preuss R, Angerer J. Di(2-ethylhexyl)phthalate (DEHP): human metabolism and internal exposure-- an update and latest results. Int J Androl. 2006;29(1):155-65; discussion 81-5. Epub 2006/02/10. doi: 10.1111/j.1365-2605.2005.00607.x. PubMed PMID: 16466535.

12.	Petroff RL, Cavalcante RG, Colacino JA, Goodrich JM, Jones TR, Lalancette C, et al. Developmental exposures to common environmental contaminants, DEHP and lead, alter adult brain and blood hydroxymethylation in mice. Front Cell Dev Biol. 2023;11:1198148. Epub 2023/06/29. doi: 10.3389/fcell.2023.1198148. PubMed PMID: 37384255; PubMed Central PMCID: PMCPMC10294071.

13.	Chang JW, Lee CC, Pan WH, Chou WC, Huang HB, Chiang HC, et al. Estimated Daily Intake and Cumulative Risk Assessment of Phthalates in the General Taiwanese after the 2011 DEHP Food Scandal. Sci Rep. 2017;7:45009. Epub 2017/03/23. doi: 10.1038/srep45009. PubMed PMID: 28327585; PubMed Central PMCID: PMCPMC5361203.

14.	Neier K, Cheatham D, Bedrosian LD, Dolinoy DC. Perinatal exposures to phthalates and phthalate mixtures result in sex-specific effects on body weight, organ weights and intracisternal A-particle (IAP) DNA methylation in weanling mice. J Dev Orig Health Dis. 2019;10(2):176-87. Epub 2018/07/12. doi: 10.1017/s2040174418000430. PubMed PMID: 29991372; PubMed Central PMCID: PMCPMC6329673.

15.	Ren WQ, Liu N, Shen Y, Wang XY, Zhou Q, Rui C, et al. Subchronic exposure to di-(2-ethylhexyl) phthalate (DEHP) elicits blood-brain barrier dysfunction and neuroinflammation in male C57BL/6J mice. Toxicology. 2023;499:153650. Epub 2023/10/20. doi: 10.1016/j.tox.2023.153650. PubMed PMID: 37858774.
